# Presence of resveratrol in wild *Arachis* species adds new value to this overlooked genetic resource

**DOI:** 10.1038/s41598-020-68648-1

**Published:** 2020-07-30

**Authors:** Paula Andreá Sampaio de Vasconcelos Carvalho, Márcio de Carvalho Moretzsohn, Ana Cristina Miranda Brasileiro, Patrícia Messenberg Guimarães, Tânia da Silveira Agostini-Costa, Joseane Padilha da Silva, Marcos A. Gimenes

**Affiliations:** 1 Universidade Estadual Paulista (UNESP), Botucatu, SP 18618-687 Brazil; 2 Embrapa Recursos Genéticos E Biotecnologia, Parque Estação Biológica, Brasília, DF 70770-917 Brazil

**Keywords:** Plant breeding, Plant breeding

## Abstract

Genus *Arachis* comprises 82 species distributed into nine taxonomic sections. Most *Arachis* species are wild and those from *Arachis* section have been evaluated for many traits, since they can be used in peanut breeding. Most of the remaining species have been neglected and understudied. Recently, resveratrol content and expression of a resveratrol synthase gene were analyzed in wild *Arachis* species. Our aim was to expand the knowledge about resveratrol in *Arachis*, analyzing species from five sections and evaluating the expression of a resveratrol synthase (RS) gene responsive to ultraviolet light (UV) along the time. In a first experiment, the resveratrol content after UV induction was analyzed on detached leaves of 12 species from five sections. Variation was observed among species and accessions of the same species. The highest contents were found in *A. lignosa* (843.9 μg/g) and *A. triseminata* (745.4 μg/g)*.* In a second experiment, RS expression and resveratrol content in four species and one synthetic amphidiploid were analyzed at 0, 7, 15 and 24 h pos induction (hpi) with UV. In most genotypes, the highest RS expression level was at 0 hpi, whereas the highest resveratrol content was at 15 hpi. Our results suggested that resveratrol is ubiquitously present in the genus *Arachis* with different capacities of synthesis among species and accessions in response to ultraviolet treatment. Presence of resveratrol in wild *Arachis* species adds new value to these genetic resources.

## Introduction

The genus *Arachis* comprises 82 species distributed into nine taxonomic sections^1-4^. *Arachis hypogaea* (cultivated peanut) is the only economically important species of the genus and is largely used as a source of oil and proteins for humans and feed stock in tropical and subtropical areas^5^. In addition, *A. pintoi* and *A. glabrata* are grown as tropical forage^6,7^ and *A. repens* is used as an ornamental plant^8^. On a limited scale, *A. villosulicarpa* and *A. stenosperma* are still cultivated by indigenous population in Brazil for food and medicinal purposes^9^.

Section *Arachis* is the most characterized section of the genus, as it comprises *A. hypogaea* and its wild relatives that have been used for the genetic improvement of the crop. The characterization of the genus has included studies on genetic diversity and phylogenetic analysis, and evaluation of different species for their resistance to several biotic and abiotic stresses^10,11^. Species from genus *Arachis* are among the few plant species that naturally synthesize resveratrol, a phytoalexin that protects plants against biotic and abiotic stresses^12–14^. Resveratrol is also well known for its potent antioxidant properties, and as a therapeutic agent in the prevention or treatment of many human diseases, including neoplastic, metabolic, cardiovascular, pulmonary and neurological disorders^15–17^. Nonetheless, to date little it is known about resveratrol synthesis and metabolic production in *wild Arachis* species.

The first study on resveratrol in wild *Arachis* comprised 10 species of section *Arachis* harboring different genome types (five A-type, three B-type, and two of the K-genome type)^18^. This study demonstrated that accessions of two species (*A. kuhlmannii* and *A. cardenasii*) produced more resveratrol than one acession of *A. hypogaea* after UV radiation, suggesting that wild *Arachis* species could be used as source of alleles for the improvement of the resveratrol content in cultivated peanut. Also, the resveratrol content and resveratrol synthase (RS) expression were studied in four *Arachis* genotypes (two wild diploid species and two allotetraploids)^19^ showing significant variation in RS expression and resveratrol content among samples. Both studies evaluated leaves after 15 h of UV induction, since previous data showed that the highest levels of resveratrol in *A. hypogaea* were observed between 12^20^ and 16 h post induction (hpi)^21^. Recently, resveratrol was also detected in two species of the section *Caulorrhizae*, *A. pintoi*^22^ and *A. repens*^23^.

The present study broadened the knowledge on resveratrol synthesis and metabolism in genus *Arachis,* as it describes the resveratrol content in species of five *Arachis* taxonomic sections (*Arachis*, *Caulorrhizae*, *Extranervosae*, *Procumbentes* and *Triseminatae*) and the expression profile of a resveratrol synthase (RS) gene and the corresponding metabolite in five wild *Arachis* species.

## Results and discussion

### Resveratrol content variation among accessions of 12 *Arachis* species

The UV induction was used to guarantee that detectable levels of resveratrol would be found. Different methods of UV induction have been patented for increasing accumulation of resveratrol in fruits and vegetables^24^. The average resveratrol contents found in leaves of 17 accessions of 12 *Arachis* species after 15 hpi with UV radiation are presented in Table 1, Experiment 1. The metabolite concentration ranged from 67.0 μg/g in *A. monticola* (section *Arachis*) to 843.9 μg/g in *A. lignosa* (section *Procumbentes*). Traces of resveratrol (below 0.1 μg/g) were detected in the controls (non-UV exposed) for all the 17 accessions (data not shown). Resveratrol has been detected in 100 species from 35 taxonomic families being the lowest content found in *Veratrum nigrum* (1 μg/g) and the highest in seeds of *Paeonia suffruticosa* var. *papaveracea* (870 μg/g)^25^. The mean resveratrol content in the most used plant for resveratrol extraction (*Polygonum cuspidatum*) is 524 μg/g in roots^26^. In this study after UV induction, we found five accessions of five species (*A. glandulifera, A. palustris, A. praecox, A. lignosa*, and *A. triseminata*) with higher resveratrol content than *Polygonum cuspidatum,* suggesting those *Arachis* species have potential to be used for resveratrol extraction. Agronomical evaluation of these accessions would be necessary to evaluate their real potential to be cultivated for commercial purposes.

**Table 1:** *Arachis* genotypes analyzed, their genome type and content of resveratrol and resveratrol synthase (RS) expression estimated after UV exposure. Means followed by the same letter do not differ (α < 0,05) according to Scott-Knott test.

Species	Section	Accession	Genome	Hours post induction collection points (h)	Resveratrol content (μg/g)	RS expression
**Experiment 1**						
*A. glandulifera*	*Arachis*	V 13738	D	15	525.2 ± 192.0^b^	–
*A. hypogaea*	*Arachis*	cv. IAC Caiapó	AB	15	273.2 ± 72.5^d^	–
		cv. Runner IAC 886	AB	15	430.7 ± 181.7^c^	–
*A. hoehnei*	*Arachis*	V 9140	A	15	108.5 ± 55.8^e^	–
		V 9094		15	68.1 ± 40.5^e^	–
*A. krapovickasii*	*Arachis*	W 1291	K	15	429.9 ± 137.3^c^	–
*A. magna*	*Arachis*	V 13765	B	15	176.7 ± 21.7^e^	–
		V 14724		15	185.0 ± 41.2^e^	–
		V14727		15	299.6 ± 93.1^d^	–
		V 14750		15	305.1 ± 86.5^d^	–
*A. monticola*	*Arachis*	V 14165	AB	15	67.0 ± 14.8^e^	–
*A. palustris*	*Arachis*	V 14156	G	15	614.9 ± 194.2^b^	–
*A. praecox*	*Arachis*	V 14682	G	15	567.3 ± 157.2^b^	–
*A. pintoi*	*Caulorrhizae*	GK 12787	C	15	302.1 ± 89.0^d^	–
*A. villosulicarpa*	*Extranervosae*	V 8816	Ex	15	241.5 ± 60.8^d^	–
*A. lignosa*	*Procumbentes*	V 13570	PR	15	843.9 ± 163.3^a^	–
*A. triseminata*	*Triseminatae*	GK 12881	T	15	745.4 ± 195.9^a^	–
**Experiment 2**						
*A. duranensis*	*Arachis*	V 14167	A	0	18.5 ± 4.3^c^	3,306.83
				7	162.5 ± 45.2^b^	514.22
				15	293.3 ± 49.7^a^	72.77
				24	362.5 ± 129.5^a^	42.10
*A. ipaënsis*	*Arachis*	K 30076	B	0	18.1 ± 4.6^d^	2,894.64
				7	209.0 ± 61.1^c^	745.59
				15	250.2 ± 64.5^b^	110.81
				24	394.7 ± 146.3^a^	375.51
*A. hypogaea*	*Arachis*	cv. Runner IAC 886	AB	0	10.0 ± 2.5^c^	1,146.38
				7	139.5 ± 44.1^b^	2,182.24
				15	331.0 ± 26.07^a^	159.08
				24	338.8 ± 73.7^a^	51.24
*A. stenosperma*	*Arachis*	V 10309	A	0	6.9 ± 1.7^c^	3,828.14
				7	176.6 ± 60.7^b^	453.59
				15	293.5 ± 46.7^a^	74.35
				24	323.1 ± 51.8^a^	136.54
(*A*. *ipaënsis x A. duranensis*)^4x^	*Arachis*	K 30076 × V 14167	AB	0	15.0 ± 2.15^b^	497.97
				7	156.5 ± 59.0^a^	35.33
				15	198.8 ± 56.2^a^	11.10
				24	251.3 ± 70.5^a^	14.43

Collectors: G = W.C. Gregory; K = A. Krapovickas; V = J.F.M. Valls.

Resveratrol was found in leaves of all species (Table 1). Resveratrol in wild *Arachis* species has been evaluated so far in a restrict number of tissues, such as leaves exposed to UV treatment^18,19^ and extracts obtained from seeds and calli^22^. However, resveratrol might also be found in other tissues, as observed in *A. hypogaea*^20^. In addition, similarity of an RS gene sequence among *A. hypogaea* and wild species was observed when primers designed for *A. stenosperma* were successfully used to amplify this RS gene on *A. hypogaea* and on the wild diploid species *A. duranensis* (A genome) and *A ipaënsis* (B genome)^19^.

**Figure 1:**
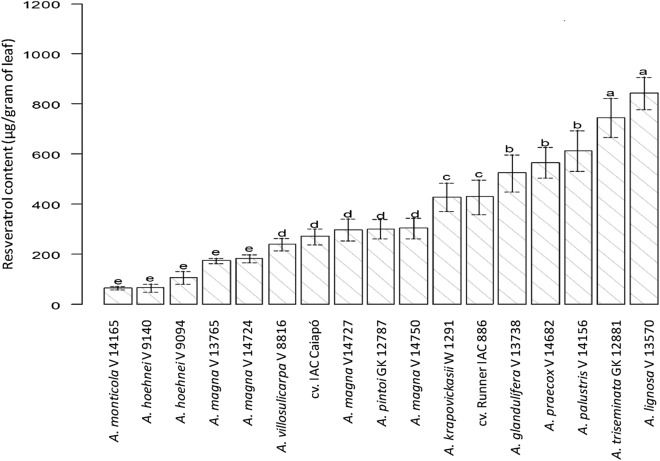
Resveratrol content in 17 accessions of 12 species of *Arachis,* after UV radiation exposure for 2h30min. Means followed by the same letter do not differ (α < 0.05) according to Scott-Knott test.

The 17 accessions analyzed in Experiment 1 were classified into five groups according to their resveratrol contents (Fig. 1): a) *A. lignosa* and *A. triseminata*; b) *A. palustris*, *A. praecox*, and *A. glandulifera*; c) *A. hypogaea* cv. Runner IAC 886 and *A. krapovickasii,* d) *A. magna* (V 14750 and V 14727), *A. hypogaea* cv. IAC Caiapó, *A. pintoi* and *A. villosulicarpa;,* e) *A. magna* (V 14724 and V 13765), *A. hoehnei* (V 9140 and V 9094) and *A. monticola.*

The “a” group (accessions GK 12881 of *A. triseminata,* and V 13570 of *A. lignosa*) exhibited the highest level of resveratrol among the 17 accessions analyzed. These values are higher than the ones found in other studies that analyzed resveratrol production under UV stimulation in peanut cultivars^50^, *Arachis* wild species^18,19^ and grape^27^. *Arachis triseminata* is not cultivated but it has a great forage potential. Its geographic distribution is restricted to a limited area in caatinga^1^, which is the most important biome for the livestock in the Brazilian semi-arid region^28^. On the other hand, the use of *A. triseminata* for the peanut pre-breeding is very limited as section *Triseminatae* is genetically distant from the *Arachis* sections^29,30^. The use of *A. lignosa* in peanut breeding is also limited and fertile hybrids resulting from crosses between species of *Arachis* and *Procumbentes* sections have not been reported.

A total of 16 types of genome have been described for the genus *Arachis,* according to cross-compatibility, chromosome morphology and cytogenetic analyses^11,31–34^. Here, the resveratrol content was analyzed on species with 10 genome types (Table 1). In general, accessions with the same genome type were spread into different groups with distinct ranges of resveratrol content (Table 1; Fig. 1). The exceptions were *A. palustris* (614.9 μg/g) and *A. praecox* (567.3 μg/g) that were located in the same significance group and both have a G genome^34^. Overall, data suggested no relationship between genome type and resveratrol content, in agreement with a previous study, which included ten *Arachis* species with four genome types (K, A, B and AB)^18^.

Variation was found among the tetraploid genotypes (Table 1). The cultivars Runner IAC 886 and IAC Caiapó had mean resveratrol concentrations of 430.7 μg/g (group c) and 273.2 μg/g (group d), respectively, while *A. monticola* produced the lowest concentration (67.0 μg/g) in this study (Table 1, Fig. 1). *Arachis monticola* and *A. hypogaea* are tetraploid species from section *Arachis* and very closely related^35,36^. The metabolic analysis of *Arabidopsis thaliana* and *Pyrus communis* var. *sativa* showed that a small number of metabolites differed in their concentrations between diploids and synthetic tetraploids for both species^37^. It was also observed that the somatic chromosome doubling of a wild diploid potato species (*Solanum bulbocastanum*) did not change the foliar metabolic profile, as the tetraploid genotypes showed similar or lower metabolite contents in comparison to their diploid progenitor^38^. Conversely, polyploids obtained by treating *Echinacea purpurea* diploid explants with colchicine presented higher biomass yield and bioactive compounds in relation to the diploid samples^39^. In *Arachis*, although not observed here (Table 1, Experiment 2), recent studies showed that synthetic allotetraploids produced more photosynthetic pigments^40^ and resveratrol^41^ than their diploid parents.

Three species had more than one accession analyzed. The two accessions of *A. hoehnei* were located in group e, the two cultivars of *A. hypogaea* in groups c and d, while two accessions of *A. magna* were placed in group d and two accessions in group e (Fig. 1). These results suggested that intraspecific genetic variability might be one of the causes for the variation on resveratrol content. Considering that genetic variability is high within species of *Arachis*^42,43^, we believe a broader study within each species will allow the identification of accessions with high resveratrol content.

Besides genetic variability, variation in resveratrol production could also be explained by differences in the plant ages and conditions in which the experiments were carried out, since many physiological and environmental factors can influence the synthesis of secondary metabolites^44–46^, such as resveratrol. These factors can probably explain the differences on resveratrol content found for IAC Caiapó here and in a previous study that also analyzed leaves after UV exposure^18^. The results of experiments 1 and 2 described here were not compared because of the different conditions they were conducted, which could be the causes of variation.

*Arachis pintoi* (*Caulorrhizae*) was placed into group d with four other accessions, with an intermediary resveratrol content. Despite of not having the highest content, *A. pintoi* is a potential source of raw material for resveratrol extraction since it has been widely cultivated as animal tropical forage, as an ornamental plant, for soil containment and for recovery of degraded areas^47,48^. Many accessions of *A. pintoi* and *A. repens*, the other species of *Caulorrhizae*^1^, are available in the Brazilian germplasm collection. The molecular characterization of these two species showed great genetic variability^49^.

Resveratrol was found in five (*Arachis*, *Caulorrhizae, Extranervosae, Procumbentes, Triseminatae*) of the nine sections of genus *Arachis*. Section *Arachis* had eight species analyzed and the other four only one accession of one species. This compound was found just recently in wild species from *Arachis* sections ^18,19^ and before that resveratrol synthesis had been reported only in the cultivated peanut. Section *Arachis* comprises 31 species^51^ and 22 of them had their resveratrol content evaluated in this and previous studies^18,19,41^. Secondary metabolite profiles of species from three major plant families (*Fabaceae*, *Solanaceae* and *Lamiaceae*) were very similar in members of monophyletic clades^52^. Thus, data obtained with section *Arachis* suggest that resveratrol will also be found in all species of the sections that had only one species analyzed in this study.

### Resveratrol synthase expression and resveratrol content analysis

**Figure 2:**
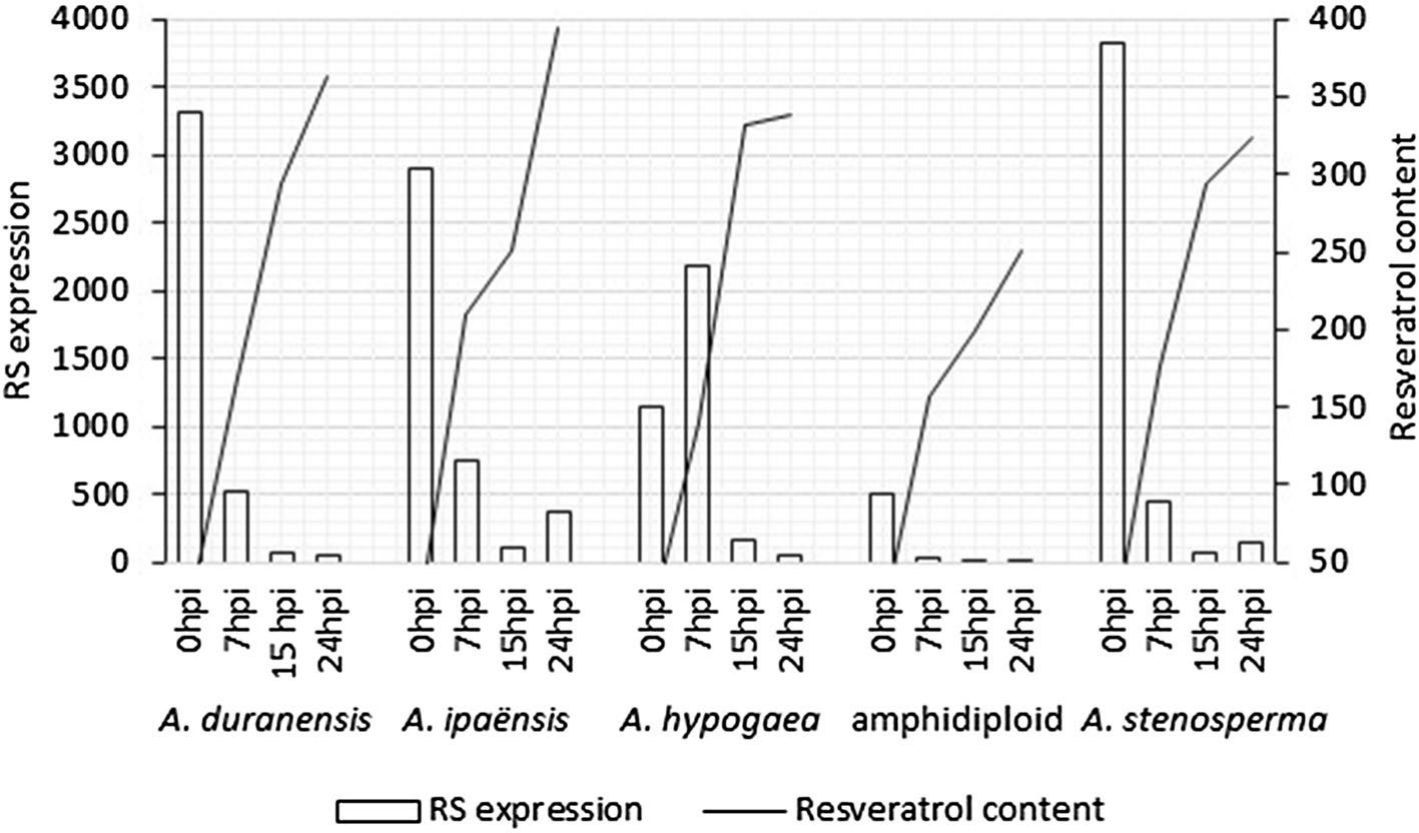
Resveratrol concentration and the relative expression of RS in each of the four collection times in relation to the control. Overall, the data suggest that there is an inverse relationship between resveratrol concentrations and RS transcripts.

We also analyzed the *Arachis* RS gene RSArAs02^19^ expression patterns and resveratrol accumulation in leaves of five *Arachis* genotypes (*A. duranensis, A. ipaënsis, A. stenosperma, A. hypogaea* cv. Runner IAC 886, and a synthetic allotetraploid) submitted to UV irradiation at four times after induction (Table 1, Experiment 2). The non-treated control samples, collected before UV induction, did not present detectable concentrations of resveratrol (data not shown) with the methodology used. We observed a general induction of RS transcripts immediately after the UV exposure (0 and 7 hpi) followed by a decrease in RS expression and an increased accumulation of resveratrol in the subsequent times (15 and 24 hpi), regardless the *Arachis* genotype. Parallel accumulation of RS mRNAs and resveratrol was observed in *A. hypogaea* after UV irradiation, suggesting a transcriptional control of the resveratrol synthesis^20,21^. Our data also suggested a transcriptional control of the RS gene but through a negative feedback since the highest RS expression and the lowest resveratrol content were observed in most samples at 0 hpi (Fig. 2). Recently, it was shown in grapevine that the application of exogenous resveratrol induced the expression of a transcription factor (*VvWRKY8*) that physically interacts with other transcription factor *(VvMYB14)*, preventing it from binding to the promoter of the RS gene *VvSTS15/21* to stimulate its expression^53^. However, if the *Arachis* orthologs of *VvWRKY8* and *VvMYB14* are also involved in this regulatory loop of resveratrol biosynthesis in the genus is yet to be clarified. In grapevine, at least 33 potentially functional RS genes show high levels of conserved gene structure^54^. Despite the conserved structure, RS grapevine genes have different patterns of transcript accumulation^55^. In peanut, four RS genes catalyze resveratrol synthesis and their up-regulation is positively correlated with an increase in resveratrol content^26^.Thus, the differences found in the RS expression patterns found in this study and previous ones might be due to the analysis of different RS genes.

The overall behavior of RS expression profile and resveratrol accumulation in response to the UV treatment were quite similar on the five *Arachis* genotypes evaluated here, although some species-specific differences were observed in a time-dependent manner. These differences might have been caused by variability in the constitutive and induced responses to UV among species. *Arabidopsis thaliana* accessions grown on lower latitudes were found to switch-on UV defenses more readily than those of higher latitudes^56^. The evaluation of sensitivity to UV-B of 140 *Glycine* spp. accessions showed intraspecific variability in the response to the radiation and the DNA sequence comparison between the most resistant and the most sensitive genotypes allowed the identification of candidate genes involved in UV-B resistance mechanism^57^.

In a previous study^19^, it was hypothesized that the variation in resveratrol content and RS expression among two wild *Arachis* species and two amphidiploids analyzed at 15 hpi with UV were due to differences in the time of response to the stimulus that resulted in an early or late increase in the resveratrol content. That hypothesis was based on the assumption that the synthesis of resveratrol after UV treatment in wild *Arachis* species was similar to its synthesis in cultivated peanut that has the highest RS expression and resveratrol content levels at the same time after induction with UV^20,21^. The data obtained in the present study demonstrated that RS gene expression peaked at 0 hpi in four of the five samples, decreasing until 15 hpi. The expression at 24 hpi varied among the samples, decreasing in *A. hypogaea* and *A. duranensis* and increasing in the synthetic amphidiploid, *A ipaënsis* and *A. stenosperma* (Fig. 2). However, those differences were not reflected in the concentrations of resveratrol, which were significantly the same at 15 and 24 hpi in most samples. The exception was *A. ipaënsis* that showed a significant increase of resveratrol and RS expression at 24 hpi. Therefore, there was no early or late response but just a differential potential among the samples analyzed of synthesizing resveratrol under UV induction conditions used in this study.

Our findings revealed that, for the five *Arachis* genotypes studied, the maximum of resveratrol leaf contents were reached between 15 and 24 h after the UV treatment indicating that 15 hpi was a good choice as the time of collection of UV-treated leaves for analyzing the resveratrol content in the 12 *Arachis* species.

## Conclusions

Our results showed that 12 species of different sections of the genus *Arachis* have the potential to produce resveratrol in response to UV radiation exposure, and there was intra and interspecific variation in the resveratrol production. That suggests that a more comprehensive study, including more sections, species, and accessions could still identify novel important sources of resveratrol in the genus*.* In addition, this study contributed to highlight the importance of the characterization and maintenance of wild species, such as *A. lignosa* and particularly *A. triseminata* that harbors high levels of resveratrol and has potential as tropical forage. To date, the cultivated species (*A. hypogaea*) was the only species of the genus considered for resveratrol production, but our data opens the possibility to species *of Caulorrhizae, Extranervosae, Procumbentes, Triseminatae* sections to be also considered for that purpose. Coupled RS expression and resveratrol content analyzes at four times post UV induction showed that all species have a similar behavior and that 15 h pos-induction was a good choice to collect material for resveratrol extraction.

## Methods

### Plant material

Twenty-two *Arachis* genotypes (21 accessions and 1 synthetic amphidiploid) were analyzed. In the first experiment, the resveratrol content was determined in 17 accessions of 12 species from five sections of the genus *Arachis* (Table 1). In a second experiment, the expression of a resveratrol synthase gene and the resveratrol content were analyzed in five genotypes (Table 1). Seeds were obtained from the *Arachis* Germplasm Active Bank of Embrapa Genetic Resources and Biotechnology, Brasília, Brazil.

Plants were cultivated between March and June (2013) for the first experiment and between October and November (2014) in a greenhouse with monitored conditions of pest control, humidity, and fertilization. The experiments were carried out at Embrapa Genetic Resources and Biotechnology, Brasília, DF, Brazil.

### Induction of resveratrol synthesis by UV

Resveratrol induction was performed based on the methodology previously used for *Arachis* species^18^. Briefly, leaves from 2-month-old greenhouse-grown plants were randomly placed into two trays containing a layer of germination paper covered with a cotton layer moistened with 500 ml of water. The tray containing the treated samples was immediately placed in a laminar flow chamber (Model FLV Series: 235–81, Trox, Brazil) and exposed to ultraviolet light (UV-C) (Philips TUV 30 W / 630 TB Longlife Lamp) for 2 h 30 min, at a distance of 50 cm from the lamp. The tray containing the control samples was left during the same time in a separated room, free of radiation. After the UV exposure, both trays were maintained in the dark at room temperature for additional 15 h for the determination of resveratrol content in 17 accessions from 12 species (Table 1, Experiment 1) and for 0, 7, 15 and 24 h for the material used for RNA and resveratrol extraction (Table 1, Experiment 2). Leaves of each genotype were divided into three aliquots of 1 g to technical repetitions and stored into 50 ml tubes wrapped in aluminum foil to protect samples from light. The experiment was repeated three times under the same conditions (biological triplicates) at intervals of 7 days.

#### Resveratrol extraction

The resveratrol extraction methodology was described by Potrebko & Resurreccion^58^, with modifications^18^. Samples were protected from light during all steps of the extraction and analyzed in technical triplicates. Ten ml of ethanol 80% (v/v) and 0.84 ml of phenolphthalein (Sigma-Aldrich) were immediately added into each tube after the maceration (1 g of leaves) using a glass stick and liquid nitrogen. Extraction was performed using a tissue homogenizer (Polytron®, Kinematica) for 2 min at 20,000 rpm. The samples were centrifuged at 10,000 rpm at 25^o^ C for 10 min and the liquid phase was transferred to another tube. Five ml of ethanol 80% (v/v) were added to each tube, vortexed for 2 min and centrifuged again. This procedure was repeated three times. Twenty ml of hexane (Merck) were added to each tube. After manual agitation, tubes were allowed to stand in the bench for one minute for the two phases separation. The upper phase was carefully discarded using a Pasteur pipette. The extract cleaning procedure was repeated once using 10 ml of hexane. The solvent was evaporated in hot plate (60^o^ C) and nitrogen gas jet (1 h 30 min). Aluminum foil wrapped flasks containing the dried residue were stored for 24 h at -20^o^ C for analysis by HPLC (High Performance Liquid Chromatography).

#### HPLC analysis

Prior to HPLC injection, the dried residue was reconstituted with 6.8 ml of ethanol. The vial was vortexed for 1 min to detach completely the extract from the tube wall and then sonicated in an ultrasonic washing (Unique USC 2880A 37 kHz) for 4 min. The procedure was repeated once to ensure the complete recovery of the extract. The sample was then transferred to a 2 ml microtube and centrifuged at 13,400 rpm for 15 min at 25° C. The supernatant was transferred to a new 2 ml microtube and used for injection to HPLC analysis that was carried out in a ProStar Varian system equipped with a ternary pump, autosampler, PDA detector, and Galaxie PS-335/Software 1.9. The column used was the Agilent Zorbax XDB C18 (250 × 4.6 mm, 5 μm), with no guard column. A gradient of acetonitrile and 0.02% aqueous phosphoric acid solution (J.T.Baker) was used as mobile phase: acetonitrile 0 min at 13%; 6–9 min at 15%; 17 min at 17%; 28–33 min at 28%; 40 min at 50%; 45 min at 60%; 46–48 min at 80%; 49–54 min at 13%; flow rate of 1.0 ml/min. UV absorption was monitored at 308 nm, 280 nm and also at the maximum absorption length of each eluent (PDA). The injection volume of the sample was 10 μl. The peak of resveratrol in the extract was identified using methodology previously described^42^. Final concentrations of resveratrol were calculated according to Potrebko & Resurreccion^58^.

The resveratrol content values are the means of three biological replicates, being each repetition analyzed by HPLC in three technical repetitions. The mean resveratrol content values were compared through variance analysis, assigning random effect among the groups of plants over time (blocks), in order to filter the observed variability due to repetitions. The Scott Knott test (α < 0.05) was used to compare the production of resveratrol among samples. The analysis was performed using the statistical language program R, free for download at https://www.r-project.org/.

### RS expression and resveratrol content coupled analysis

#### RNA extraction

Total RNA was extracted from *Arachis* leaves using a protocol described previously^59^. For that, leaf samples were pooled at equal amounts per biological replicate to form three pooled UV-treated samples and three pooled non-treated control samples. Each pooled sample (1 g) was triturated in liquid nitrogen and 900 μl of preheated CTAB extraction buffer (2% CTAB; 2% PVP; 100 mM Tris–HCl pH 8.0; 25 mM EDTA; 1,4 M NaCl; 0.5 g/L-spermidine and 0.2% beta-mercaptoethanol) were added to each tube. The tubes were vortexed and incubated at 60° C for 10 min and 700 μl of chloroform:isoamyl alcohol (24:1) were added to each tube, mixed and centrifuged at 13,000 rpm for 10 min at 4° C. The supernatant was transferred to a new tube and the procedure repeated once. LiCl solution (4 M) was added to each tube (¼ of total volume). After 2 h, the samples were centrifuged at 13,000 rpm for 30 min at 4° C. The total RNA was washed by adding 500 μl of 70% ethanol and centrifuged at 13,000 rpm for 10 min at 4° C. The supernatant was discarded and dried at room temperature for 30 min. The pellet was resuspended in 20 μl of autoclaved DEPC-treated (diethylpyrocarbonate) water.

The amount of total RNA extracted was estimated at 260 nm using the NanoDrop ND-1000 spectrophotometer (Thermo Scientific; Wilmington, USA). The integrity was evaluated using 1.5% agarose gel electrophoresis (1% TAE buffer at 60 V, for approximately 1 h) and ethidium bromide staining.

#### DNAse treatment and cDNA synthesis

Total RNA (2 μg) was purified using the Invinb® Spin Plant RNA Mini Kit (Invitek; Berlin, Germany) and treated with 2 U of deoxyribonuclease (TURBO™ Dnase Applied Biosystem; Foster City, CA, USA) at 37 °C for 1 h to eliminate possible contamination with genomic DNA. After incubation, DNAse was inactivated by adding 2.5 mM EDTA to each sample and incubating for 10 min at 65° C, according to manufacturer’s instructions. The cDNA was then synthesized as previously described^60^.

Genomic DNA contamination in cDNA samples was further checked by RT-PCR using a pair of primers from the Actin (ACT2) that flank an intronic region in *Arachis* spp.^61^. The RT-PCR product was then analyzed by electrophoresis as described above.

#### qRT-PCR

Reactions were performed in a 7,300 Real Time PCR system (Applied Biosystem; Foster City, USA) using 5 μl of Platinum SYBR Green qPCR Super Mix -UDG w/ROX kit (Invitrogen, Carlsbad, CA, USA); 0.2 mM of RSArAs02 primers^19^ and 2 μl of 100X diluted cDNA in a final volume of 10 μl^19^. The genes of *Arachis* spp. coding for the 60S ribosomal protein (60S) and ubiquitin (UBI2) were used as reference genes for normalization of expression values^61^.

Three independent biological replicates were analyzed per sample and the average cycle threshold (Cq) values were estimated using the online real-time PCR Miner tool^62^. The relative expression of transcripts from the UV-treated (UV) samples relative to the non-treated control (CTR) samples were determined and statistically tested using REST 2009 software^63^.
